# Anti-Cancer Activity of Maize Bioactive Peptides

**DOI:** 10.3389/fchem.2017.00044

**Published:** 2017-06-21

**Authors:** Jorge L. Díaz-Gómez, Fabiola Castorena-Torres, Ricardo E. Preciado-Ortiz, Silverio García-Lara

**Affiliations:** ^1^Agri-Foods Unit, Tecnologico de MonterreyMonterrey, Mexico; ^2^Tecnologico de Monterrey, Escuela de MedicinaMonterrey, Mexico; ^3^Maize Breeding Program-INIFAP Campo Experimental BajíoCelaya, México

**Keywords:** maize, disease, peptides, bioactive, antioxidant, anticancer, antihypertensive, hepatoprotective

## Abstract

Cancer is one of the main chronic degenerative diseases worldwide. In recent years, consumption of whole-grain cereals and their derivative food products has been associated with a reduced risk of various types of cancer. The main biomolecules in cereals include proteins, peptides, and amino acids, all of which are present in different quantities within the grain. Some of these peptides possess nutraceutical properties and exert biological effects that promote health and prevent cancer. In this review, we report the current status and advances in knowledge regarding the bioactive properties of maize peptides, such as antioxidant, antihypertensive, hepatoprotective, and anti-tumor activities. We also highlight the potential biological mechanisms through which maize bioactive peptides exert anti-cancer activity. Finally, we analyze and emphasize the potential applications of maize peptides.

## Introduction

According to the World Health Organization, chronic diseases are currently the major cause of morbidity worldwide, and will become one of the major causes of mortality by the next decade (WHO, 2017). In recent years, the consistent consumption of cereals and cereal-derived food products has been linked with a reduced risk of cancer and other chronic degenerative diseases. Several reports indicate that diets rich in whole-grain cereals are associated with lower cancer mortality rates, particularly colon, breast, and prostate cancers. (Jeong et al., 2003; Liu, 2007).

Cereals contain nutraceutical molecules that can exert specific biological effects and promote health and prevent diseases (Chaturvedi et al., 2011). These biomolecules are proteins and their derivatives, peptides, and amino acids present in different quantities in the grain. Legumes such as soybean are the most studied source of bioactive proteins and peptides due to their high (up to 40%) average protein content (Cavazos and Gonzalez de Mejia, 2013). Cereals such as wheat, rice, barley, rye, and maize have been recently identified as new sources of bioactive peptides. High quality cereal proteins are an important source of bioactive peptides, which consist of distinctive amino acid sequences, and which, once they are released, could display diverse functionalities (de Mejia et al., 2012; Zambrowicz et al., 2013). Cereal bioactive peptides have been a part of the human diet for centuries. In addition to their nutritional roles, these peptides could perform biological activities (Dia and Mejia, 2010).

The potential therapeutic use of peptides derived from food has been discussed previously (Malaguti et al., 2014; Ortiz-Martinez et al., 2014), and these peptides have displayed a broad range of effects in different models (McDermott, 2009). The main objective of this review was to report the current status and advances in knowledge regarding the bioactive properties of maize peptides, and to review the evidence relating to the identification, characterization, and relevance of the biological activities through which these peptides exert an anti-cancer effect.

## Maize as a source of bioactive peptides

Cereals, which are members of the grass family *Gramineae*, are the most important source of foods for the world population, and the main source of carbohydrates, proteins, vitamins, and minerals. Maize, rice, and wheat are the most important grains in the human diet, and with more than 1,000 million tons harvested in 2014, maize is the most popular crop worldwide (FAO, 2015).

A maize kernel consists of an embryo (or germ), an endosperm packed with starch grains, and bran (fiber). The most abundant kernel nutrient is starch, which is composed mainly of amylopectin and amylose (72–73% of the total kernel weight), followed by proteins that represent ~8–12% of the total kernel weight (FAO, 1992). The essential amino acid content is ~5–10% lower than the non-essential amino acid content, with glutamic acid being the most abundant amino acid (Tang et al., 2013). Four groups of storage proteins are present in maize kernels: albumins, globulins, prolamins, and glutelins. Albumins and globulins are found mainly in the germ, while prolamins and glutelins are found predominantly in the endosperm (Shukla and Cheryan, 2001). These four classes are also categorized according to their solubility: water-soluble albumins, globulins soluble in salt solution, prolamins soluble in alcoholic, and glutelins insoluble in neutral aqueous or saline solutions and ethanol. Globulins and albumins regulate and control grain metabolism, whereas prolamins and glutelins store the nitrogen necessary for seed germination (Anderson and Lamsal, 2011).

**Figure 1:**
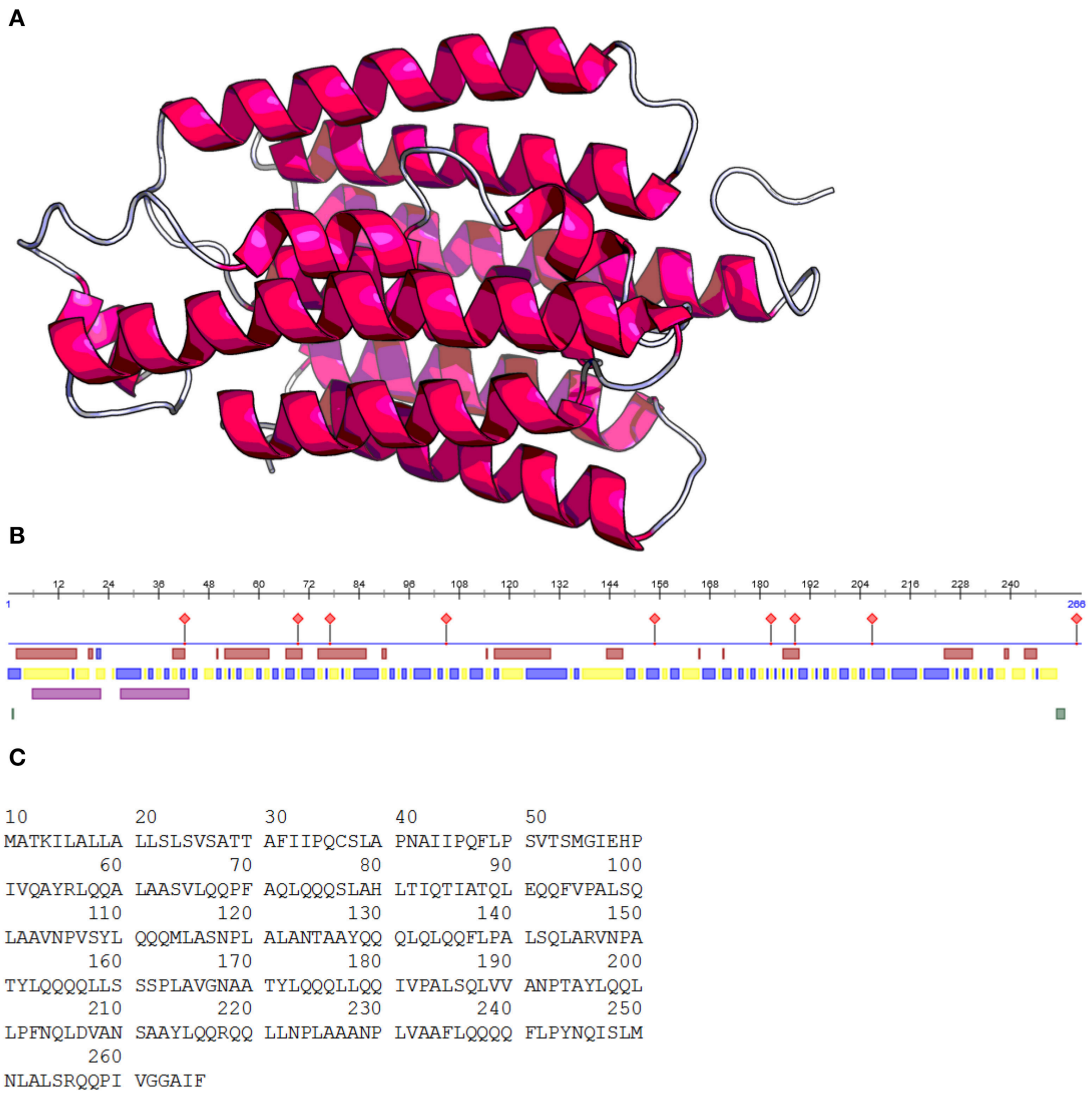
Structure prediction of α-zein 22 kDa **(A)**. Zein regions **(B)**: protein binding regions (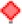), helix (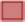), exposed region (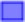), buried region (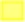), helical transmembrane region (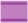), disordered region (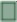). Zein amino acid sequence **(C)**. Images obtained using prediction web programs PredictProtein and RaptorX (Rost, 2003; Källberg et al., 2012).

In terms of quantity, the protein content of maize kernels is composed mostly of prolamins or zeins (40%), followed by glutelins (30%), with globulins and albumins found in lesser quantities (5%) (Wang et al., 2008). Zeins are mainly found in protein bodies in the rough endoplasmic reticulum and constitute ~44–79% of maize endosperm proteins (Giuberti et al., 2012). Zeins are devoid of lysine and tryptophan, amino acids that are essential for human survival (Huang et al., 2004). Zeins are composed of four fractions: α, γ, β, and δ; α-zeins represent 71–85% of the prolamins in the grain, whereas γ-, β-, and δ-zeins represent 20, 5, and 5% of the prolamins in the grain, respectively. Therefore, α-zein is the most important fraction because it stores most of the nitrogen (Momany et al., 2006). A structural representation and the amino acid sequence of α -zein are shown in Figure 1. The molecular weights of the four fractions are as follows: α, 19 and 22 kDa; γ, 18 and 27 kDa; β, 16 kDa; and δ, 10 kDa (Anderson and Lamsal, 2011). Another α zein fraction with a molecular weight of 24 kDa contains a defective signal peptide that induces the incorrect formation of zein bodies in the cell. This variant is present in the floury-2 (*fl2*) maize mutants that have higher content of the essential amino acid lysine in the endosperm, and this mutation leads to the synthesis of the α-zein variant and the defective signal peptide (Coleman et al., 1995). The most abundant amino acids found in these fractions are glutamic acid, leucine, proline, and alanine (Kong and Xiong, 2006).

Albumins are richer in aspartate, asparagine, threonine, glycine, alanine, proline, and half-cystine, but have low histidine, arginine, glutamic acid, glutamine, and phenylalanine content (Landry and Moureaux, 1987). Glutelins are alkali-soluble proteins with molecular weights of 10, 15, 18, and 27 kDa; they are present in protein bodies along with zeins, and their amino acid composition and function is similar to that of zeins (Wall et al., 1988).

### Bioactive peptides properties

In general, cereal-derived peptides have been shown to possess opiate, antithrombotic, anticancer, antihypertensive, immunomodulatory, mineral-binding, antimicrobial, and antioxidant properties (de Mejia et al., 2012). Various bioactive peptides have been found and reported in maize (Tnani et al., 2013; Li et al., 2014). Maize bioactive peptides are obtained by the hydrolysis of kernels and sub-products. Among the different techniques for peptide generation, enzymatic hydrolysis is the most widely used method for maize. The main enzymes used in the hydrolysis of maize protein are listed in Table 1. For bioactivity evaluations, peptides have been fractionated by ultra-filtration (Wang Y. et al., 2014; Jin et al., 2016), and separated using chromatographic techniques with multiple purification steps (Puchalska et al., 2013; Wang X.-J. et al., 2014; Jin et al., 2016; Wang et al., 2016) to obtain low molecular weight peptides that have shown promise in further characterization. Various bioactive properties of maize peptides have been reported, including anticancer activity and other activities that have beneficial effects on health, such as antioxidant, antihypertensive, hepatoprotective, and alcohol protective activities. The different studies reporting the bioactive properties of maize peptides are listed in Table 1.

**Table 1:** Main bioactivities of maize peptides, and methods used for their enzymatic hydrolysis fractionation.

**Type of bioactivity**	**Bioactivity measured**	**Enzyme used**	**Use of ultrafiltration/chromatografy**	**References**
Anti-Oxidant	Inhibition of pyrogallol oxidation	Alcalase	N/Y	Zheng et al., 2006
	Inhibition of lipid peroxidation, reducing power, scavenging activity	Alcalase	N/Y	Li et al., 2008
	Radical scavenging, removal of superoxide anion, inhibition of lipid peroxidation, reducing power	Alcalase	N/Y	Li et al., 2010
	Radical scavenging	Alcalase	Y/Y	Tang et al., 2010
	Scavenging activity	None	N/N	Zhang et al., 2011
	Oxygen radical absorbance, scavenging activity, chelating activity, inhibition of lipid peroxidation	Neutral protease, alkaline protease, protease validase	Y/N	Zhou et al., 2012
	Scavenging activity of different radicals, chelating activity, inhibition of lipid peroxidation	Alkaline protease, flavourzyme	Y/Y	Zhuang et al., 2013
	Scavenging activity, chelating activity, reducing power,	Alkaline protease, trypsin, papain, flavourzyme	Y/Y	Tang and Zhuang, 2014
	Scavenging activity, chelating activity, reducing power	Alcalase	Y/Y	Wang X.-J. et al., 2014
	Scavenging activity, reducing power	Alkaline protease	Y/Y	Zhou et al., 2015
	Scavenging activity, oxygen radical absorbance, antioxidant effect in HepG2 and Caco2 cells	Alcalase	Y/N	Wang et al., 2015
	Scavenging activity, chelating activity, reducing power	Alcalase, flavourzyme	Y/Y	Jin et al., 2016
	Scavenging activity, inhibition of aggregation and oligomerization of Aβ peptides in *Caenorhabditis elegans*	Alcalase	N/Y	Zhang et al., 2016
Anti-Hypertensive	Inhibitory activity of ACE, decrease of blood pressure in an animal model	Trypsin	Y/Y	Yang et al., 2007
	Inhibitory activity of ACE	Trypsin, thermolysin, flavourzyme, fungal protease	Y/N	Parris et al., 2008
	Inhibitory activity of ACE, decrease of blood pressure in an animal model	Alcalase	Y/N	Huang et al., 2011
	Inhibitory activity of ACE, decrease of blood pressure in an animal model	Alcalase	Y/Y	Lin et al., 2011
	Inhibitory activity of ACE	Neutral protease	N/N	Zhou et al., 2013
	Inhibitory activity of ACE	Thermolysin	Y/Y	Puchalska et al., 2013
Hepato-Protective	Changes in hepatic enzyme levels, histopathological changes in hepatic tissue	Alcalase, neutral protease	N/N	Guo et al., 2009
	Changes in hepatic enzyme levels, histopathological changes in hepatic tissue	Alcalase	Y/N	Yu et al., 2012
	Decrease in alcohol blood concentration in an animal model	Alcalase	Y/Y	Ma et al., 2012
	Decrease in alcohol blood concentration in an animal model, changes in hepatic enzyme levels	Alcalase	Y/N	Yu et al., 2013
	Changes in hepatic enzyme levels, histopathological changes in hepatic tissue,	Alcalase	Y/Y	Lv et al., 2013
	Changes in hepatic enzyme levels in a clinical trial	Alcalase	Y/N	Wu et al., 2014
	Changes in hepatic enzyme levels, inhibition of apoptosis	Alcalase	Y/N	Ma et al., 2015
Anti-Cancer	Apoptosis induction in a HepG2 cell line, reduction of hepatic tumor growth in an animal model, stimulation of the immune system	Alcalase	Y/N	Li et al., 2013
	Antiprofilerative, modulation of cell cycle and apoptosis induction in a HepG2 cell line.	Alcalase	Y/Y	Ortiz-Martinez et al., 2017
Antimicrobial	Growth inhibition *in vitro* in bacetria and fungi (Inhibition of spore germination, hyphal elongation)	Trypsin, endoproteinase Glu-C, thermolysin	Y	Duvick et al., 1992

## Health effects of maize peptides

### Anticancer

In the past few years, an important advance in cereal peptides research has been the finding that novel cereal-derived proteins and peptides exert preventive effects in different stages of cancer, including initiation, promotion, and progression. Because of the urgent need for effective cancer prevention therapy, chemoprevention has emerged as a viable anti-cancer approach. Chemo-preventive agents are expected to be safe, inexpensive, and abundant. Peptides fulfill these criteria, and are considered to be safer than synthetic compounds as they are present in the regular human diet and have a wide range of availability and acceptability (Li et al., 2014). Several studies have shown the anti-cancer potential of dietary proteins, peptides, and amino acids in the regulation of apoptosis and angiogenesis, important steps in controlling tumor metastasis; these molecules are naturally occurring or generated by fermentation, enzymatic hydrolysis, or gastrointestinal digestion (de Mejia and Dia, 2010).

Bioactive peptides exert anti-tumor activity via several key mechanisms: (a) Apoptosis induction, which involves an energy-dependent cascade mediated via specific proteases or caspases; strategies to overcome tumor resistance to apoptotic pathways include activation of pro-apoptotic receptors, restoration of p53 activity, caspase modulation, and proteasome inhibition (Burz et al., 2009). (b) Blockade of intermediate tumor generation by regulating cellular mechanisms associated with cell proliferation and survival, or biosynthetic pathways that control cell growth (Kornienko et al., 2013). (c) Regulation of immune system function by increasing the expression of tumor-associated antigens (antigenicity) in cancer cells, by triggering tumor cells to release danger signals that stimulate immune responses (immunogenicity), or by increasing the predisposition of tumor cells to be recognized and killed by the immune system (susceptibility) (Zitvogel et al., 2013).

The anticancer activities of different peptides have been characterized, with most of them showing pro-apoptotic activity (Dia and Mejia, 2010; Gonzalez de Mejia et al., 2010; Dia and Gonzalez, 2011; McConnell et al., 2015). Some mechanisms proposed for the anticancer activity of peptides are autophagy and apoptosis (de Mejia and Dia, 2010; Hernandez-Ledesma et al., 2013), and maize peptides may promote these processes in different cancer cells.

A primary anticancer effect of maize peptides has been demonstrated using *in vitro* models. HepG2 cells showed an increase in apoptotic activity when they were exposed to maize peptides obtained by enzymatic hydrolysis of protein extracted from corn gluten meal (Li et al., 2013). In that study, maize peptides were also evaluated in H22-tumor bearing mice. Treatment of animals with these peptides at a dose of 400 mg/kg resulted in an inhibition of tumor growth. Moreover, the administration of maize peptides enhanced immune system activity compared with that in the control group.

More recently, a study by Ortiz-Martinez et al. (2017) revealed important anti-cancer effects of maize peptides in HepG2 cells. Peptide fractions isolated from albumin Alcalase hydrolysates from normal maize were stronger than the ones from quality protein maize. The treatment of HepG2 cells with this peptide fraction from the different maize varieties increased the apoptosis induction rates an average of 4-fold. These results suggested that the antiproliferative effect of peptide fractions isolated from both varieties was based on the induction of apoptosis due to the decrease in antiapoptotic factor expression.

### Antioxidant

Antioxidant capacity is the main reported biological activity of maize peptides. This activity is explained by the presence of specific amino acids with radical scavenging and reducing capacities, such as lysine, tyrosine, phenylalanine, proline, alanine, histidine, and leucine (Zhou et al., 2015). Several peptides with antioxidant activity have been identified; however, their antioxidant potential depends on the enzymatic process used to obtain them. Zheng et al. (2006) studied the antioxidant capacity of peptides obtained using an optimized enzymatic hydrolysis process. Peptides with low molecular weight (<5 kDa) exhibited higher antioxidant activity (Li et al., 2008; Zhou et al., 2012; Zhuang et al., 2013; Tang and Zhuang, 2014). The properties of these small peptides included hydroxyl radical and free radical scavenging activity, inhibition of lipid peroxidation, and ion chelating capacity. The antioxidant potential is not altered in peptides obtained from total protein or zein fraction (Li et al., 2010; Tang et al., 2010). Another factor affecting antioxidant activity is the pH: native α-zein has more antioxidant activity than α-zein extracted in less basic or acidic conditions (Zhang et al., 2011). Synthesized peptides also show similar antioxidant activities (Wang X.-J. et al., 2014; Jin et al., 2016).

Additionally, peptides smaller than 3 kDa have shown potent antioxidant activity in *in vitro* assays in HepG2 and Caco2 cells exposed to high oxidative stress (Wang et al., 2015). Maize peptides also enhance the activity of cellular enzymes, and therefore, may exert preventive effects against cell damage via their antioxidant activity (Wang et al., 2016). Furthermore, in studies conducted in macrophage models, maize peptides have shown anti-inflammatory effects (Hernández-Ledesma et al., 2009; Cam and Gonzalez, 2012), which could have been exerted via their antioxidant properties. Oxidative stress has been shown to induce lipid peroxidation, protein oxidation, and DNA damage, subsequently causing mutant cell proliferation and finally, carcinogenesis (Thanan et al., 2014). These properties are relevant because of the relationship between oxidative stress and degenerative processes like carcinogenesis. Therefore, the antioxidant activity of maize peptides could be beneficial for cancer treatment.

### Antihypertensive

Maize peptides have shown antihypertensive effects. A single peptide with an Ala–Tyr sequence, obtained from corn gluten meal, which is a main by-product of maize wet milling, showed significant inhibitory activity against angiotensin I-converting enzyme (Yang et al., 2007; Lin et al., 2011). Moreover, the same peptide has shown antihypertensive activity in spontaneously hypertensive rats with a minimum effective oral dose of 50 mg/kg resulting in a diastolic blood pressure reduction of 9.5 mm Hg (Yang et al., 2007), and with an oral dose of 450 mg/kg resulting in a reduction of 40 mmHg (Lin et al., 2011). In another report, peptides smaller than 3 kDa showed greater blood pressure reductions (up to 34.45 mmHg) at a dose of 100 mg/kg in a spontaneously hypertensive rat model (Huang et al., 2011). These results show that molecular weight influences antihypertensive activity, with peptides smaller than 1 KDa being the most active. In addition, enzymes used for protein hydrolysis and peptide generation also influence the antioxidant activity of the resulting peptides, with peptides obtained using trypsin and thermolysin being the most potent (Parris et al., 2008). In addition, ultrasonic treatment applied to corn gluten meal proteins before hydrolysis appears to increase the antihypertensive activity of the resulting peptides (Zhou et al., 2013). Antihypertensive peptides have been isolated and identified as LRP (leucine–arginine–proline), LSP (leucine–serine–proline), and LQP (leucine–glutamine–proline) by using high-performance liquid chromatography (Puchalska et al., 2013). Hence, molecular interactions of peptides can affect enzymes responsible for vascular hemodynamics, suggesting further potential applications for these enzymes.

### Hepatoprotective

Recent studies have shown that maize peptides exert a protective effect by reducing damage to hepatic tissue. Using rats with liver damage induced by exposure to lipopolysaccharides from bacillus Calmette-Guérin, Guo et al. (2009) showed that administration of 600 mg/kg peptide significantly lowered the level of cell necrosis, hepatic lesions, and enzymes indicative of liver damage compared to that in control rats. In this study, the peptides protective effect on liver cells could be attributable to their antioxidant capacity. Maize peptides smaller than 5 kDa exerted a hepatoprotective effect in a mouse model of carbon tetrachloride-induced hepatic damage. At a dose of 200 mg/kg, these peptides altered hepatic enzyme levels, lowering aspartate transaminase and alanine transaminase, and elevating superoxide dismutase and glutathione (Yu et al., 2012). Maize peptides may also exert a hepatoprotective effect by facilitating alcohol metabolism. Peptides as small as *five* amino acids have been shown to display this activity in mice (Ma et al., 2012). This effect is explained by the presence of leucine, which maintains the tricarboxylic acid cycle by supplying NAD+ (antioxidant effect). The same small peptide shown anti-apoptotic activity in mouse liver cells treated with alcohol to induce damage (Ma et al., 2015). A similar study found a hepatoprotective effect in mice at a dose of 200 mg/kg with peptides smaller than 5 kDa, which resulted in lower blood alcohol concentrations (Yu et al., 2013). Maize peptides have also shown protective activity against hepatic fibrosis. In rats exposed to thioacetamide, which induces hepatic fibrosis, the minimum protective dose was 100 mg/kg (Lv et al., 2013). These studies demonstrate the hepatoprotective effect and mechanisms of action of maize peptides in response to different types of stress, suggesting another possible field of therapeutic application.

### Other properties

In addition to the effects discussed so far, a few more bioactive properties of maize peptides have been identified. Maize basic peptide 1 (BMP-1), which was obtained through protein hydrolysis, identified, and later synthetized, was shown to exert antimicrobial activity, inhibiting the growth of fungi and bacteria (Duvick et al. 1992). In an *in vivo* study in *Caenorhabditis elegans*, Zhang et al. (2016) demonstrated that a bioactive maize tetrapeptide possessed scavenging activity against intracellular reactive oxygen species. Additionally, this tetrapeptide inhibited the aggregation and oligomerization of β-amyloid peptide, which are involved in the development of Alzheimer's disease. These studies indicate the different bioactivities of maize peptides, which warrant further study and may be beneficial in the treatment of different diseases.

## Therapeutic perspectives and conclusions

Peptides have attracted attention as drug candidates because they offer certain key advantages over alternative molecules. In contrast to traditional drugs, peptides have high affinity, strong specificity, low toxicity, and adequate tissue penetration. The therapeutic use of peptides has remained limited because of their high instability in biological environments, rapid depuration from the blood, poor membrane transportability, and effective digestion in the gastrointestinal tract (Sarmadi and Ismail, 2010). Peptide-based therapy depends on the ability of the peptide to remain intact until it reaches the target organ. Bioactive peptides must remain active and intact during gastrointestinal digestion and absorption to reach the cardiovascular system and potentially exert their physiological effects (Bhutia and Maiti, 2008).

Evidence supports the use of maize peptides as therapeutic molecules against a broad array of diseases linked to oxidative damage, such as cancer. *In vitro* models have been useful to investigate the antihypertensive and anticancer effects of maize peptides. However, in addition to *in vitro* evidence, *in vivo* experiments and clinical trials are needed to demonstrate the physiological effects of peptides. Few clinical trials have been conducted involving peptides and cancer (Gustafsson et al., 2004). Some synthetized peptides derived from seaproducts are being tested in phase II trials, these include: BioPep, Plitidepsin, Elisidepsin, and Tasidotin (Bouglé and Bouhallab, 2017). Even so this studies indicate that there is an enormous potential in the bioactivity of peptides derived from foods for the prevention and/or treatment of cancer. Also, there is an important opportunity for maize peptides with anticancer activity in diverse cancer cell lines as well as in different animal models that represent different carcinomas. In a future, the clinical efficacy will likely require intervention at several levels, and is necessary to test to evaluate their safety in short and long-term *in vivo* models and clinical trials on a large and heterogeneous populations.

## Author contributions

All authors contributed equally to this work in terms of writing and conception. All authors wrote and reviewed the latest version of this manuscript.

### Conflict of interest statement

The authors declare that the research was conducted in the absence of any commercial or financial relationships that could be construed as a potential conflict of interest.
